# Immunoglobulin E levels and pregnancy-induced hypertension: Japan Environment and Children’s Study

**DOI:** 10.1038/s41598-021-88227-2

**Published:** 2021-04-21

**Authors:** Hyo Kyozuka, Tsuyoshi Murata, Toma Fukuda, Yuta Endo, Akiko Yamaguchi, Shun Yasuda, Aya Kanno, Akiko Sato, Yuka Ogata, Mitsuaki Hosoya, Seiji Yasumura, Koichi Hashimoto, Hidekazu Nishigori, Keiya Fujimori, The Japan Environment and Children’s Study (JECS) Group, Michihiro Kamijima, Shin Yamazaki, Yukihiro Ohya, Reiko Kishi, Nobuo Yaegashi, Chisato Mori, Shuichi Ito, Zentaro Yamagata, Hidekuni Inadera, Takeo Nakayama, Hiroyasu Iso, Masayuki Shima, Youichi Kurozawa, Narufumi Suganuma, Koichi Kusuhara, Takahiko Katoh

**Affiliations:** 1 Department of Obstetrics and Gynecology, Fukushima Medical University School of Medicine, 1 Hikarigaoka, Fukushima, 960-1295 Japan; 2 Fukushima Regional Center for the Japan Environmental and Children’s Study, Fukushima Medical University School of Medicine, 1 Hikarigaoka, Fukushima, 960-1295 Japan; 3 Department of Pediatrics, Fukushima Medical University School of Medicine, 1 Hikarigaoka, Fukushima, 960-1295 Japan; 4 Department of Public Health, Fukushima Medical University School of Medicine, 1 Hikarigaoka, Fukushima, 960-1295 Japan; 5 Fukushima Medical Center for Children and Women, Fukushima Medical University School of Medicine, 1 Hikarigaoka, Fukushima, 960-1295 Japan; 6 Graduate School of Medical Sciences Department of Occupational and Environmental Health, Nagoya City University, 1 Kawasumi, Mizuho-cho, Mizuho-ku, Nagoya, Aichi 467-8601 Japan; 7 National Institute for Environmental Studies, Tsukuba, Japan; 8 National Center for Child Health and Development, Tokyo, Japan; 9 Hokkaido University, Sapporo, Japan; 10 Tohoku University, Sendai, Japan; 11 Chiba University, Chiba, Japan; 12 Yokohama City University, Yokohama, Japan; 13 University of Yamanashi, Chuo, Japan; 14 University of Toyama, Toyama, Japan; 15 Kyoto University, Kyoto, Japan; 16 Osaka University, Suita, Japan; 17 Hyogo College of Medicine, Nishinomiya, Japan; 18 Tottori University, Yonago, Japan; 19 Kochi University, Nankoku, Japan; 20 University of Occupational and Environmental Health, Kitakyushu, Japan; 21 Kumamoto University, Kumamoto, Japan

**Keywords:** Immunology, Health care, Risk factors

## Abstract

High serum immunoglobulin E (IgE) levels are associated with cardiovascular events. We aimed to evaluate the association between total IgE levels during the first trimester of pregnancy and pregnancy-induced hypertension (PIH) development in a large Japanese cohort. We analysed data pertaining to singleton primipara pregnancies recorded in the Japan Environment and Children’s Study involving births from 2011 to 2014. Serum IgE levels were determined using the immunonephelometric technique. High serum IgE was defined as level ≥ 170 IU/ml. Hypertensive disorders in pregnancy (HDP) were categorized into early onset (Eo) PIH (developed < 34 weeks) or late onset (Lo) PIH (developed ≧ 34 weeks). A multiple logistic regression model was used to estimate the risk of high serum IgE levels on PIH, Eo-PIH, and Lo-PIH. Overall, 32,518 participants were enrolled. The prevalence of total, Eo-, and Lo-PIH was 3.2%, 0.6%, and 2.3%, respectively. Patients with high serum IgE levels had an increased risk of Lo-HDP (adjusted odds ratio [aOR]:1.19, 95% confidence interval 1.01–1.40). No correlation was found with either PIH (total) or Eo-PIH. High serum IgE levels during the first trimester were associated with the risk of Lo-PIH. Our results could influence and shape further research regarding the pathogenesis of Lo hypertension.

## Introduction

Hypertensive disorders of pregnancy (HDP) occur in approximately 2.5% of all pregnancies in Japan^1^. HDP is a public health burden, because it is the direct cause of approximately 30,000 maternal deaths per year and accounts for 14% of maternal deaths worldwide^2,3^. Pre-eclampsia (PE) and gestational hypertension (GH) are the main forms of HDP, together referred to as pregnancy-induced hypertension (PIH), which are conventionally defined as new onset hypertension after 20 weeks of gestation, with (in cases of PE) or without (in cases of GH) signs of dysfunction in organs including the kidney, liver, and placenta^4^. PIH can also be categorised into early-onset (Eo)-PIH (onset hypertension before 34 weeks) and late-onset (Lo)-PIH (onset of hypertension after 34 weeks)^5^. These conditions have different clinical implications. Eo-type but not Lo-type is a high-risk factor for foetal death (adjusted odds ratio [aOR], 5.8; 95% confidence interval [CI] 4.0–8.3 vs aOR 1.3; 95% CI 0.8–2.0, respectively)^6^. However, both Eo- and Lo-type result in 10- and twofold increase in perinatal maternal death, respectively, compared with that in pregnant women without newly onset hypertension during pregnancy^5^. Although there are distinct perinatal differences between Eo-PIH and Lo-PIH, the exact mechanisms leading to Eo- and Lo-PIH remain unknown. The clinical burden of PIH necessitates that the mechanisms underlying the pathophysiology of PIH are elucidated in order to implement preventative strategies.

Mast cells are essential components of asthma and allergic responses^7,8^. One of the best-known mechanisms for mast cell activation is the binding of immunoglobulin E (IgE) to its high-affinity receptor FceR1 on the mast cell surface. After IgE binding, mast cells release histamine, mast cell protease, proteoglycan, cytokines, and chemokines^9,10^. Many of these inflammatory mediators are associated with the development of pregnancy-related hypertension^11–14^. Therefore, high serum IgE levels during early pregnancy could be associated with later maternal cardiovascular events such as PIH. Nevertheless, little is known about the relationship between serum total IgE levels during the first trimester of pregnancy and the development of PIH.

Therefore, the aim of this study was to evaluate the association between serum IgE levels during the first trimester and the development of PIH, including both Eo- and Lo-PIH, using nationally representative data from the Japan Environmental and Children's Study (JECS)^15^.

## Results

**Figure 1:**
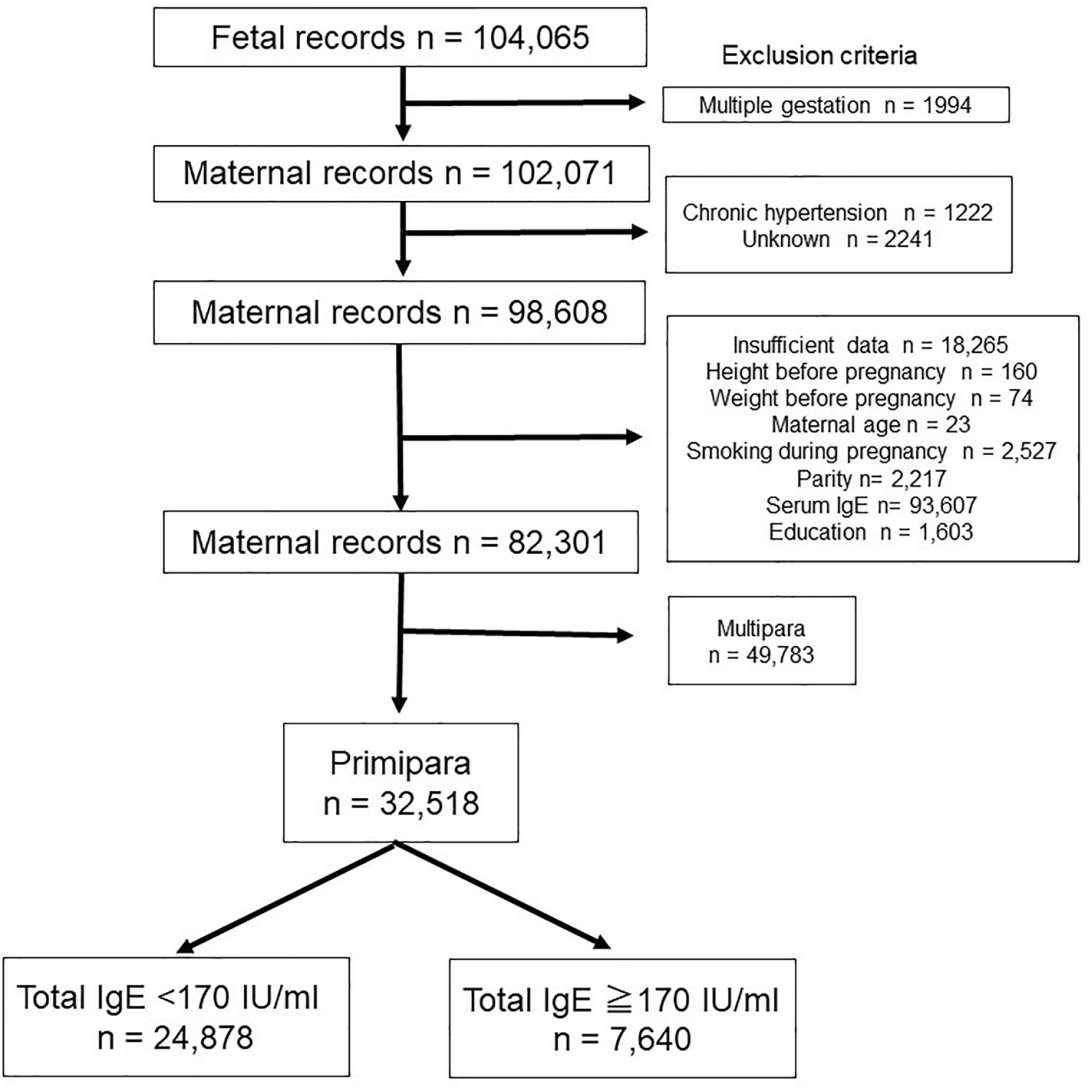
Flow chart of the study participants. *IgE* immunoglobulin E.

**Figure 2:**
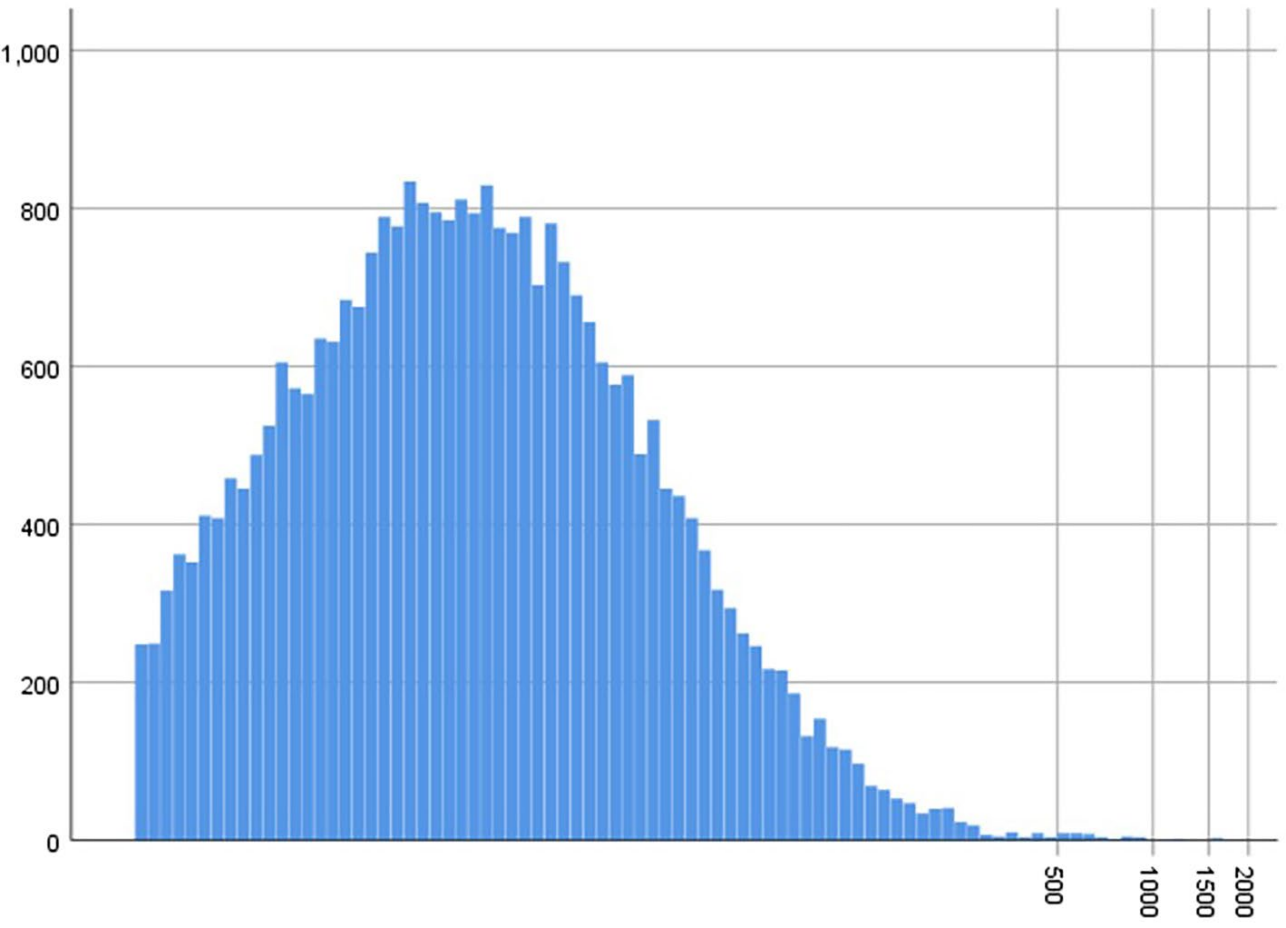
Concentration of the total serum IgE level. The horizontal axis indicates the log-transformed total serum IgE level, and the vertical axis indicates the number of participants.

In the JECS dataset, the total number of records for infants delivered between 2011 and 2014 was 104,065. Of these, 1994 infants were delivered as a result of multiple gestation, 1222 participants had maternal chronic hypertension, 16,307 participants had insufficient data, and 49,783 were multiparous participants, and they were excluded. As a result, 32,518 participants who were singleton primipara and had no history of hypertension before pregnancy were eligible for enrolment in this study. They were subsequently categorised into two groups according to positive IgE sensitisation (IgE < 170 IU/ml or IgE ≥ 170 IU/ml) (Fig. 1). The median [interquartile (IQR)] serum IgE level for all participants was 60.0 (range 21.5–159.0) IU/ml. The concentration of the total serum IgE level is presented in Fig. 2. Because the value of serum IgE skewed to the right, the total serum IgE level was transformed into a logarithmic scale on the horizontal axis. In total, 1067 cases (3.2%) developed PIH (Eo-PIH: 196 cases (0.6%), Lo-PIH: 769 cases (2.3%)). For 102 cases of PIH, the gestational age at onset of hypertension was unknown.

### Maternal medical and socioeconomic background and obstetric outcomes

The maternal characteristics and obstetric factors were summarised according to the serum IgE levels and Table 1 summarises the maternal medical background data and the obstetric outcomes for the groups classified according to the IgE levels (IgE < 170 or IgE≧170 IU/ml). The mean gestational age at blood sample collection was not significantly different between two groups (p = 0.068). The mean maternal age significantly differed between the two groups (p < 0.01), with women in the IgE < 170 IU/ml group being older. Because the main outcome in this study was new onset hypertension during pregnancy, we compared the maternal blood pressure between two groups during early trimester. The mean gestational age at the time of measurement of maternal blood pressure was also not significantly different between two groups (p = 0.068). Neither the mean maternal systolic nor the diastolic blood pressure measured during blood sample collection was remarkably different (mean, standard deviation [SD], 111 mmHg^15^ vs 111 mmHg^12^, p = 0.232; and 64 mmHg^13^ vs 64 mmHg^14^, p = 0.098; respectively). Body mass index (BMI) categories and maternal education levels were significantly different between the participants in the two groups (p < 0.001 and p < 0.001, respectively), with the participants in the IgE ≥ 170 IU/ml group having a higher BMI and lower educational level. The ratio of smokers was higher in the ≥ 170 IU/ml group than in the IgE < 170 IU/ml group (4.8% and 3.2%, respectively, p < 0.01). There was no significant difference with respect to prevalence of systemic lupus erythematosus (SLE) (0.1% and 0.1%, p = 0.698) between the groups. Regarding the obstetric outcomes, no pertinent differences were found in the development of PIH (p = 0.114), Eo-PIH (p = 0.351), and Lo-PIH (p = 0.058).

**Table 1:** Maternal background and obstetric outcomes based on serum IgE levels.

Variable	Participants		p-value
	Total IgE < 170 IU/ml	Total IgE ≥ 170 IU/ml	
	n = 24,878	n = 7640	
**Maternal medical background**			
Gestational age at the time of blood sample collection, mean weeks (SD)	15.6 (3.2)	15.6 (3.3)	0.068^a^
Maternal age, mean years (SD)	30.1 (5.1)	29.3 (5.2)	< 0.01^a^
**Maternal age category, %**			
≦19	1.3	2.4	< 0.01^b^
20–29	46.1	51.3	
30–39	48.7	43.7	
≥ 40	4.0	2.6	
Timing at measure maternal blood pressure, weeks mean (SD)	11.1 (1.8)	11.1 (1.8)	0.300^a^
Systolic blood pressure in the first trimester, mean mmHg (SD)	111 (15)	111 (12)	0.232^a^
Diastolic blood pressure in the first trimester, mean mmHg (SD)	64 (13)	64 (14)	0.098^a^
**BMI before pregnancy (kg/m**^**2**^**), %**			
< 18.5	17.7	16.9	< 0.01^b^
18.5–25.0	74.0	73.4	
> 25.0	8.3	9.7	
Smoking during pregnancy, %	3.2	4.8	< 0.01^b^
SLE, %	0.1	0.1	0.698^b^
**Maternal education, year, %**			
< 10	3.3	4.7	< 0.01^b^
10–12	28.8	30.3	
13–16	43.0	41.1	
≥ 17	24.9	23.9	
**Obstetric outcomes**			
Gestational age at delivery, weeks mean (SD) (%)	38.7 (2.2)	38.8 (2.1)	0.016^a^
PIH	3.2	3.6	0.114^b^
Eo-PIH	0.6	0.5	0.351^b^
Lo-PIH	2.3	2.7	0.058^b^

*IgE* immunoglobulin E, *SD* standard deviation, *BMI* body mass index, *SLE* systemic lupus erythematosus, *PIH* pregnancy induced hypertension, *Eo* early-onset, *Lo* late-onset.

^a^p-value, t-test.

^b^p-value, chi-square test.

### Maternal background and obstetrics outcomes among those with Eo- and Lo-PIH

Participants were categorised into one of the three following groups, the non-PIH group (defined as control), Eo-PIH group, or Lo-PIH group. The prevalence of obstetric outcomes in PIH, including pre term birth (PTB), low birth weight (LBW), and small for gestational age (SGA), was compared across the three groups. Table 2 summarises the maternal background data and obstetrics outcomes among the control, Eo-PIH, and Lo-PIH groups. The mean maternal age (SD) among the control, Eo-PIH, and Lo-PIH groups was 29.9 (5.1), 32.1 (5.8), and 31.1 (5.4), respectively, and was found to be significantly different among the three groups (p < 0.01). There was no notable difference in gestational age among the three groups (p = 0.700).

**Table 2:** Maternal background and obstetric outcomes for Eo-PIH and Lo-PIH.

Variable	Participants			p-value
	Control n = 31,451	Eo-PIH n = 196	Lo-PIH n = 769	
**Maternal medical background**				
Maternal age, years mean (SD)	29.9 (5.1)	32.1 (5.8)	31.0 (5.4)	< 0.01^a^
**Maternal age category, %**				
≦19	1.5	0.5	1.2	< 0.01^b^
20–29	47.6	35.7	39.0	
30–39	47.3	55.1	52.7	
≥ 40	3.5	8.7	7.2	
Gestational age at blood sample, mean (SD) weeks	15.6 (3.4)	15.5 (3.5)	15.5 (3.2)	0.700^a^
Total IgE IU/ml median (inter-quartile)	60.2 (21.5–158.0)	57.4 (18.3–139.3)	62.6 (25.2–179.5)	0.190^c^
Total IgE ≥ 170 (IU/ml), %	23.4	20.9	26.5	0.090^b^
**BMI before pregnancy (kg/m**^**2**^**), %**				
< 18.5	17.7	7.7	13.0	< 0.01^b^
18.5–25.0	74.2	61.7	67.5	
> 25.0	8.2	30.6	19.5	
Smoking during pregnancy, %	3.6	4.1	3.6	0.934^b^
SLE, %	0.1	0.5	0.0	0.167^b^
**Maternal education, year, %**				
< 10	3.6	5.1	4.4	< 0.05^b^
10–12	29.1	29.6	29.3	
13–16	42.5	45.4	46.6	
≥ 17	24.8	19.9	19.8	
**Obstetric outcomes**				
PTB < 37 weeks, %	3.9	43.9	8.2	< 0.01^b^
LBW < 2500 g, %	8.5	48.7	20.5	< 0.01^b^
SGA, %	4.9	26.2	11.6	< 0.01^b^

*Eo* early-onset, *Lo* late-onset, *HDP* hypertensive disorders of pregnancy, *SD* standard deviation, *IgE* immunoglobulin E, *BMI* body mass index, *SLE* systemic lupus erythematosus, *PTB* preterm birth, *LBW* low birth weight, *SGA* small for gestational age.

^a^p-value, one-way analysis of variance.

^b^p-value, chi-square test.

^c^p-value, Kruskal–Wallis test.

The median (IQR) serum total IgE level between the control, Eo-PIH, and Lo-PIH groups was not significantly different (60.2 [21.5–158.0] IU/ml, 57.4 [18.3–139.3] IU/ml, and 62.6 [25.2–179.5] IU/ml, respectively, p = 0.190). The proportion of positive serum IgE cases in the control, Eo-PIH, and Lo-PIH groups was not also significantly different (23.4% vs 29.0% vs 26.5%, respectively, p = 0.090).

The ratio of smokers and those with systemic lupus erythematosus were not significantly different among the three groups (p = 0.934, p = 0.167, respectively). The proportion of women with a body mass index (BMI) > 25.0 kg/m^2^ and the proportion of women with an education level < 10 years were significantly higher in the Lo-PIH group (p < 0.01 and p < 0.01, respectively).

Regarding obstetrics outcomes in PIH, the PTB < 37 weeks, LBW < 2500 g, and SGA were significantly different among the three groups (p < 0.01, p < 0.01, and p < 0.01, respectively). The prevalence of PTB < 37 weeks, LBW < 2500 g, and SGA was highest in the Eo-PIH group. Overall, the incidence of these outcomes was highest in the Eo-PIH group, followed by the Lo-PIH group, and lowest in the control group.

### Association between high serum IgE during early trimester and occurrence of PIH

We calculated the adjusted odds ratio (aOR) to estimate the risk of high serum IgE during early trimester on newly onset hypertension during pregnancy. Table 3 summarises the association between serum IgE levels ≥ 170 IU/ml and the prevalence of PIH (total), Eo-PIH, and Lo-PIH. Using the IgE < 170 IU/ml group as reference, multiple logistic regression analyses were performed which showed that IgE ≥ 170 IU/ml was associated with an increased risk of developing Lo-PIH (aOR 1.19, 95% CI 1.01–1.40). However, IgE levels ≥ 170 IU/ml were not associated with the onset of either PIH (total) or Eo-PIH (aOR 1.15, 95% CI 0.99–1.32 and aOR 0.85, 95% CI 0.60–1.21, respectively).

**Table 3:** Relationship between IgE levels ≥ 170 IU/ml and HDP, Eo-HDP, and Lo-HDP.

	HDP	Eo-HDP	Lo-HDP
Number	1067	196	769
Case, %	3.2	0.6	2.3
IgE (−)	Ref	Ref	Ref
IgE (+) OR (95% CI)	1.14 (0.99–1.31)	0.86 (0.61–1.22)	1.18 (1.01–1.39)
IgE (+) aOR (95% CI)	1.15 (0.99–1.32)	0.85 (0.60–1.21)	1.19 (1.01–1.40)

aOR was calculated by logistic regression analysis, using maternal age, body mass index before pregnancy, systemic lupus erythematosus, maternal smoking status, and maternal education.

*IgE* immunoglobulin E, *HDP* hypertensive disorders of pregnancy, *Eo* early-onset, *Lo* late-onset, *OR* odds ratio, *CI* confidence interval, *aOR* adjusted odds ratio, *Ref* reference.

## Discussion

In this study, although we assessed data pertaining to maternal background (such as maternal age, BMI before pregnancy, maternal education, and smoking habit) and analysed the differences between participants with serum IgE levels < 170 IU/ml and IgE ≥ 170 IU/ml, we found no differences regarding the onset of PIH (total), Eo-PIH, and Lo-PIH between the two groups. We also compared maternal background variables with obstetric outcomes such as PTB, LBW, and SGA in those without PIH and in participants with Eo-PIH and Lo-PIH. Our findings indicated significant differences in adverse obstetrics outcomes and maternal background between the three groups. Multiple logistic regression analyses found that high serum IgE levels during first trimester were a risk factor for development of Lo-PIH.

To the best of our knowledge, only one study has examined the association between maternal serum IgE levels and obstetric outcomes. This study suggested that IgE levels in the third trimester of pregnancy and cord blood were strongly related to birth outcomes and foetal growth restriction^16^. Most previous studies that measured serum IgE levels have focused on cardiovascular events such as hypertension or coronary artery disease in the general population^17,18^. A study including 156 patients with coronary heart disease showed a significantly higher total IgE concentration in patients with unstable angina and acute myocardial infarction than in those with stable angina or those in the control group^19^. In another study, 195 patients with any form of ischemic heart disease had significantly higher total IgE levels than those in the control group^20^. Guo et al. reported that serum IgE levels were significantly higher in patients with multi-vessel disease than in those with single vessel disease, suggesting that a higher serum IgE level was an independent predictor for acquiring multi vessel disease^21^. On the contrary, a cross sectional study that included 315 women showed no significant positive correlation between increased serum IgE levels and cardiovascular disease^22^. In this study, we used the cut off for total IgE as 170 for the follow reasons: First, the participants in this study were Japanese women and Japanese commercial medical test companies set the cut off for total IgE as 170 IU/ml. Second, previous Japanese studies have also applied the cut off as 170 IU/ml for total serum IgE levels^23^. Finally, the value of total serum IgE is not normally distributed and strongly skewed to the right (Fig. 2). Therefore, we thought it would be reasonable to use a cut off value of 170 IU/ml. With regard to the educational status, our data consisted of categorical variables. With regard to BMI, we have applied this categorization many times in our previous analysis^1^ and have used a BMI of 18.5 to 25.0 as the reference range for conducting a logistic regression analysis^24–26^.

Despite evidence that high total IgE levels are associated with cardiovascular disease, an important question remains; do increased total IgE levels precede or result from cardiovascular events?

The exact cause of PIH also remains unclear. Eo-PIH is reportedly caused by the failure of the trophoblasts to invade the maternal spiral artery, resulting in a high maternal vascular resistance^27,28^. As a result, foetal growth restriction or SGA usually occurs^29,30^, frequently requiring preterm delivery for maternal and/or foetal indications. On the contrary, later newly onset of hypertension during pregnancy is considered to be more of a maternal constitutional disorder^31^ due to underlying maternal microvascular disorders or sustained maternal stress^32^, in which poor trophoblast invasion is less likely to play a significant role. Lo-PIH is more common than Eo-PIH and often has a mild course but can be associated with significant clinical morbidities^33^. Consistent with previous studies, in the present study, the prevalence of PTB < 37 weeks, LBW < 2500 g, and SGA in the Eo-PIH group (43.9%, 48.7%, and 216.2%, respectively) was higher than that in the LO-PIH group (8.2%, 20.5%, and 11.6%, respectively). High IgE levels are associated with microvascular disorders, and maternal microvascular disorders could result in Lo-PIH. Therefore, our results that maternal high serum IgE levels are a risk factor for later newly onset hypertension during pregnancy are biologically plausible.

This was the first large-scale study conducted in Japan by the Japanese government with meticulous attention to data collection. Therefore, this study is considered to be representative of the general pregnant population in Japan^34^. Additionally, we only included primipara cases and had a sufficiently large number of the same in the ethnic population. Nevertheless, this study also has potential limitations. First, although we accounted for some confounding factors in large portions of the questionnaire, unknown factors that could affect the occurrence of PIH might have existed because the pathogenesis of PIH is still to be elucidated. There are unknown factors that may change the present finding that higher maternal serum IgE levels are associated with PIH. Second, although obstetric outcomes were based on medical records, this study focused on PIH, which does not differentiate between GH and PE as we did not have information regarding the severity of hypertension, presence of urine protein, or any organ dysfunction. As PE is accompanied by foetal growth restriction, proteinuria, or other organ dysfunction, the complications of PE more severely affect the neonate than do those of GH. In addition, different pathogenesis may exist among these two phenotypes^35^. However, there is also evidence that up to 50% of women with GH will eventually develop PE^36^ and the future risk for maternal cardiovascular events such as chronic hypertension is the same^37^. Therefore, we thought GH and PE may be undistinguishable in terms of long-term maternal cardiovascular risks.

Finally, since there are very few previous studies on serum IgE levels during pregnancy^35^, the cut-off value for positive IgE (IU/ml) was defined as 170 IU/ml. The cut-off value of serum IgE levels may have affected the results of the logistic regression analysis. Thus, further studies that examine the relationship between serum IgE levels and the risk of Lo-PIH occurrence, are warranted.

Our findings indicate that high serum IgE levels (≥ 170 IU/ml) during the first trimester are associated with the risk of Lo-PIH. The results of this study may provide novel insights into the pathogenesis of new onset hypertension during pregnancy. However, only a few studies have previously measured maternal serum IgE levels, and hence additional studies are needed to confirm or refute our findings.

## Methods

### Study design

The present study utilised data from the JECS, a government-funded birth cohort study that commenced in January 2011. The JECS investigated the effects of several environmental factors, such as heavy metals and allergens, on children’s health^15^. Pregnant women were eligible for participation in the JECS: (1) if lived in the study area at the time of the application and expected to live in Japan in the near future; (2) if they had an expected delivery date between 1 August 2011 and mid-2014; and (3) if they were able to participate without difficulty (i.e. they could complete the self-management questionnaires). The JECS protocol was reviewed and approved by the Ministry of the Environment’s Institutional Review Board on Epidemiological Studies and by the Ethics Committees of all participating institutions. The study was conducted in accordance with the Helsinki Declaration and other nationally valid regulations and guidelines. Written informed consent was obtained from all participants.

### Data collection

The current analysis utilised the JECS data set released in March 2018 (data set: jecs-an-20180131), from which we used the following information: (1) a self-reported questionnaire completed in the first trimester, including details about medical conditions at the time of pregnancy, number of previous deliveries, and smoking status; (2) a self-reported questionnaire completed during the second/third trimester, which included particulars regarding socioeconomic data; (3) obstetric outcomes retrieved from medical record transcripts provided by a co-operating health care provider, and (4) maternal blood sample records, collected during the first trimester.

### Inclusion and exclusion criteria

Maternal medical conditions at the time of pregnancy include hypertension, thyroid disease, psychological disease, gastro intestinal disease, epilepsy, SLE, cancer, and diabetes mellitus. We excluded a case with hypertension at the time of pregnancy because this study’ focus was new onset of hypertension during pregnancy. We included SLE as a possible confounding factor because it has potential risk of PIH^6^.

This study only involved primipara women since the risk of PIH is much higher in them compared to multipara women^36^. In the present study, we excluded cases with insufficient data, multiple pregnancies, hypertension at the time of pregnancy, and multipara.

### Measurement of total IgE, obstetrics outcomes, and confounding factors

Blood samples were obtained from the mother during the first trimester of pregnancy. Serum total IgE titres were analysed in a contract clinical laboratory by immunological assays. Maternal serum total IgE titres were assayed by fluorescence immunoassay, using ImmunoCAP (Thermo Fisher Scientific, Inc., Sweden)^38^. A high serum IgE level was defined as IgE ≥ 170 IU/ml based on the results of a Japanese cross-sectional study in pregnant women^23^. Maternal systolic and diastolic blood pressures were measured at the time of blood sample collection.

Obstetrics outcomes included PIH, gestational age at birth, and birth weight. PIH was defined as new onset hypertension (≥ 140/90 mmHg) after conception^4^. PIH was further classified into two categories: Eo-PIH (PIH onset before 34 weeks of gestation) and Lo-PIH (PIH onset after 34 weeks of gestation). SGA was defined as a birth weight less than − 1.5 SDs below the population mean, corrected for gestational age and sex according to the ‘New Japanese neonatal anthropometric charts for gestational age at birth^39^. PTB was defined as delivery before 37 gestational weeks. LBW was defined as birth weight < 2500 g.

The following parameters were considered to be confounding factors: maternal age at delivery, BMI before pregnancy, maternal smoking status, maternal educational status, and SLE. The mothers were categorised into four age groups: < 20, 20–29, 30–39, or ≥ 40 years. The maternal BMI before pregnancy was calculated by dividing the mother’s weight (kg) by the square of the mother’s height (m). BMI was categorised into < 18.5, 18.5–25.0, or > 25.0 kg/m^2^^1^.

A self-reported questionnaire during first trimester had the following options regarding smoking history: ‘Never’, ‘Previously did. Quit prior to current pregnancy’, ‘Previously did. Quit during this pregnancy’, and ‘Currently smoking’. Women who chose ‘Currently smoking’ were considered smokers (smoking category); otherwise, they were considered non-smokers (non-smoking category).

Based on the Japanese educational system, maternal education was categorised into the following four groups: junior high school: < 10, high school: 10–12, professional school or university: 13–16, and graduate school: ≥ 17 years of education^1^.

Maternal participants were requested to provide the following information regarding SLE: ‘Have you ever been diagnosed with SLE at a medical institution?’. The maternal participants who answered ’Yes’ were classified into the SLE group^40^. The confounding factors in this study were determined based on previously identified risk factors for newly onset hypertension during pregnancy^41–44^.

#### Statistical analyses

T-test and chi-square tests were performed to compare continuous and categorical variables, respectively. To compare more than three variables, the Kruskal–Wallis (one-way analysis of variance) and chi-square tests were used. Finally, logistic regression models were used to calculate the aORs and 95% CI for PIH, Eo-PIH, and Lo-PIH. An aOR was calculated after adjusting for maternal age, BMI before pregnancy, maternal smoking during pregnancy, SLE, and maternal education. Logistic regression model analysis was performed using dummy variables for categorical variables composed of more than three categories (e.g. BMI could be categorised as < 18.5, 18.5 − 25.0, and > 25). SPSS version 26 (IBM Corp., Armonk, NY) was used for the statistical analyses. A p-value < 0.05 was deemed to be statistically significant.

## Data Availability

The datasets analysed during the current study are not publicly available due to confidentiality/research subject protections. The authors, with permission of the Eco-child Study Investigation Committee and the Japan government, can make the datasets available upon reasonable request.

## References

[1] Kyozuka H (2018). The Japan Environment and Children's Study (JECS) in Fukushima prefecture: pregnancy outcome after the great east Japan earthquake. Tohoku J. Exp. Med..

[2] Kassebaum NJ (2014). Global, regional, and national levels and causes of maternal mortality during 1990–2013: A systematic analysis for the Global Burden of Disease Study 2013. Lancet.

[3] Say L (2014). Global causes of maternal death: a WHO systematic analysis. Lancet Glob. Health.

[4] Brown MA (2018). Hypertensive disorders of pregnancy: ISSHP classification, diagnosis, and management recommendations for international practice. Hypertension.

[5] Lisonkova S (2014). Maternal morbidity associated with early-onset and late-onset preeclampsia. Obstet. Gynecol..

[6] Lisonkova S, Joseph KS (2013). Incidence of preeclampsia: risk factors and outcomes associated with early- versus late-onset disease. Am. J. Obstet. Gynecol..

[7] Theoharides TC, Kalogeromitros D (2006). The critical role of mast cells in allergy and inflammation. Ann. N. Y. Acad. Sci..

[8] Bradding P, Walls AF, Holgate ST (2006). The role of the mast cell in the pathophysiology of asthma. J. Allergy Clin. Immunol..

[9] MacGlashan D, Lavens-Phillips S, Katsushi M (1998). IgE-mediated desensitization in human basophils and mast cells. Front. Biosci..

[10] Gruber B (1989). Activation of rheumatoid synovial mast cells. Role of IgE-associated antiglobulins. Monogr. Allergy.

[11] Harmon AC (2016). The role of inflammation in the pathology of preeclampsia. Clin. Sci. (Lond.).

[12] Formby B (1995). Immunologic response in pregnancy. Its role in endocrine disorders of pregnancy and influence on the course of maternal autoimmune diseases. Endocrinol. Metab. Clin. N. Am..

[13] Hanna N (2000). Gestational age-dependent expression of IL-10 and its receptor in human placental tissues and isolated cytotrophoblasts. J. Immunol..

[14] Wegmann TG, Lin H, Guilbert L, Mosmann TR (1993). Bidirectional cytokine interactions in the maternal-fetal relationship: Is successful pregnancy a TH2 phenomenon?. Immunol. Today.

[15] Kawamoto T (2014). Rationale and study design of the Japan Environment and Children’s Study (JECS). BMC Public Health.

[16] Xiong F, Tong Y, Li P, Huo T, Mao M (2015). Serum immunoglobulin E level and its impact on the pregnancy outcome associated with fetal growth restriction: A prospective cohort study. Genet. Mol. Res..

[17] Wang Z, Shen XH, Feng WM, Qiu W (2017). Mast cell specific immunological biomarkers and metabolic syndrome among middle-aged and older Chinese adults. Endocr. J..

[18] Kounis NG, Hahalis G (2016). Serum IgE levels in coronary artery disease. Atherosclerosis.

[19] Korkmaz ME (1991). Levels of IgE in the serum of patients with coronary arterial disease. Int. J. Cardiol..

[20] Sinkiewicz W, Błazejewski J, Bujak R, Kubica J, Dudziak J (2008). Immunoglobulin E in patients with ischemic heart disease. Cardiol. J..

[21] Guo X (2016). Serum IgE levels are associated with coronary artery disease severity. Atherosclerosis.

[22] Criqui MH, Lee ER, Hamburger RN, Klauber MR, Coughlin SS (1987). IgE and cardiovascular disease. Results from a population-based study. Am. J. Med..

[23] Miyake Y (2004). Relationship between active and passive smoking and total serum IgE levels in Japanese women: Baseline data from the Osaka Maternal and Child Health Study. Int. Arch. Allergy Immunol..

[24] Kyozuka H (2021). Japan Environment and Children's Study (JECS) Group. Prepregnancy antiinflammatory diet in pregnant women with endometriosis: The Japan Environment and Children's Study. Nutrition.

[25] Kyozuka H (2020). Dietary inflammatory index during pregnancy and the risk of intrapartum fetal asphyxia: The Japan Environment and Children's Study. Nutrients.

[26] Kyozuka H (2020). Association between pre-pregnancy calcium intake and hypertensive disorders during the first pregnancy: The Japan environment and children's study. BMC Pregn.y Childbirth.

[27] Brosens I, Pijnenborg R, Vercruysse L, Romero R (2011). The, "great obstetrical syndromes" are associated with disorders of deep placentation. Am. J. Obstet. Gynecol..

[28] Kyozuka H (2020). Comprehensive metabolomic analysis of first-trimester serum identifies biomarkers of early-onset hypertensive disorder of pregnancy. Sci. Rep..

[29] Odegard RA, Vatten LJ, Nilsen ST, Salvesen KA, Austgulen R (2000). Preeclampsia and fetal growth. Obstet. Gynecol..

[30] Jelin AC (2010). Early-onset preeclampsia and neonatal outcomes. J. Matern. Fetal Neonatal Med..

[31] Raymond D, Peterson E (2011). A critical review of early-onset and late-onset preeclampsia. Obstet. Gynecol. Surv..

[32] Kyozuka H (2019). The effect of the great east Japan earthquake on hypertensive disorders during pregnancy: A study from the Fukushima health management survey. J. Matern. Fetal Neonatal Med..

[33] Kenneth L, Hall DR, Gebhardt S, Grové D (2010). Late onset preeclampsia is not an innocuous condition. Hypertens. Pregn..

[34] Yamaguchi A (2019). Risk of preterm birth, low birthweight and small-for-gestational-age infants in pregnancies with adenomyosis: A cohort study of the Japan Environment and Children's Study. Acta Obstet. Gynecol. Scand..

[35] Gyselaers W (2020). Preeclampsia is a syndrome with a cascade of pathophysiologic events. J. Clin. Med..

[36] Sibai BM, Stella CL (2009). Diagnosis and management of atypical preeclampsia-eclampsia. Am. J. Obstet. Gynecol..

[37] Williams D (2011). Long-term complications of preeclampsia. Semin. Nephrol..

[38] Yamamoto-Hanada K (2017). Allergic profiles of mothers and fathers in the Japan Environment and Children's Study (JECS): A nationwide birth cohort study. World Allergy Organ. J..

[39] Itabashi K, Miura F, Uehara R, Nakamura Y (2014). New Japanese neonatal anthropometric charts for gestational age at birth. Pediatr. Int..

[40] Murata T (2020). Risk of adverse obstetric outcomes in Japanese women with systemic lupus erythematosus: The Japan Environment and Children's Study. PLoS ONE.

[41] Sibai BM (1996). Treatment of hypertension in pregnant women. N. Engl. J. Med..

[42] ACOG Practice Bulletin No. 2020 (2019). Gestational Hypertension and Preeclampsia. Obstet. Gynecol..

[43] Bainbridge SA, Slide EH, Smith GN (2005). Direct placental effects of cigarette smoke protect women from pre-eclampsia: the specific roles of carbon monoxide and antioxidant systems in the placenta. Med. Hypotheses.

[44] Xue T, Zhu T, Lin W, Talbott EO (2018). Association between hypertensive disorders in pregnancy and particulate matter in the contiguous United States, 1999–2004. Hypertension.

